# Interference With *ACSL1* Gene in Bovine Adipocytes: Transcriptome Profiling of mRNA and lncRNA Related to Unsaturated Fatty Acid Synthesis

**DOI:** 10.3389/fvets.2021.788316

**Published:** 2021-12-16

**Authors:** Yanbin Bai, Xupeng Li, Zongchang Chen, Jingsheng Li, Hongshan Tian, Yong Ma, Sayed Haidar Abbas Raza, Bingang Shi, Xiangmin Han, Yuzhu Luo, Jiang Hu, Jiqing Wang, Xiu Liu, Shaobin Li, Zhidong Zhao

**Affiliations:** ^1^College of Animal Science and Technology & Gansu Key Laboratory of Herbivorous Animal Biotechnology, Gansu Agricultural University, Lanzhou, China; ^2^College of Animal Science and Technology, Northwest A&F University, Xianyang, China

**Keywords:** lncRNA, ACSL1, bovine adipocytes, unsaturated fatty acids, RNA-Seq

## Abstract

The enzyme long-chain acyl-CoA synthetase 1 (ACSL1) is essential for lipid metabolism. The *ACSL1* gene controls unsaturated fatty acid (UFA) synthesis as well as the formation of lipid droplets in bovine adipocytes. Here, we used RNA-Seq to determine lncRNA and mRNA that regulate UFA synthesis in bovine adipocytes using RNA interference and non-interference with *ACSL1*. The corresponding target genes of differentially expressed (DE) lncRNAs and the DE mRNAs were found to be enriched in lipid and FA metabolism-related pathways, according to GO and KEGG analyses. The differentially expressed lncRNA- differentially expressed mRNA (DEL-DEM) interaction network indicated that some DELs, such as TCONS_00069661, TCONS_00040771, TCONS_ 00035606, TCONS_00048301, TCONS_001309018, and TCONS_00122946, were critical for UFA synthesis. These findings assist our understanding of the regulation of UFA synthesis by lncRNAs and mRNAs in bovine adipocytes.

## Introduction

As a member of the ACSL family (ACSL1, 3, 4, 5, and 6), long-chain acyl-CoA synthase 1 (ACSL1) is needed for the activation, transportation, and degradation of fatty acids (FAs), as well as lipid synthesis (1, 2). ACSL1 is found on the outer mitochondrial membrane (2) and converts long-chain FAs into fatty acyl-CoA to synthesize triglycerides (3), stimulate FA deposition, activate FAs (1), and finally to enter the β-oxidation pathway (4). Previous research has found that the presence of long-chain unsaturated FAs in beef, such as DHA (22: 6, *n*−3) and EPA (20: 5, *n*−3), aids in the prevention of cardiovascular disease, atherosclerosis, and the promotion of fetal brain and visual growth (5–8). A quantitative trait locus (QTL) study revealed that *ACSL1* influences the relative content of UFAs, omega-3 FAs, polyunsaturated FAs, docosapentaenoic acid, and DHA (9). Furthermore, overexpression of *ACSL1* activates and transports FA for the synthesis of diglycerides and phospholipids rather than cholesterol esters (10). In adipocytes, the arachidonic acid levels were further stimulated by the overexpression of *ACSL1* (11). Furthermore, four transcription factors, E2F1, E2F4, Sp1, and KLF15, symbiotically control *ACSL1* expression, suggesting that *ACSL1* controls UFA synthesis in cells (12). We have also observed, using RNA interference (13) and gene overexpression (14), that *ACSL1* controlled UFA synthesis and the formation of lipid droplets in bovine adipocytes.

Long non-coding RNAs (lncRNAs) are noncoding RNA molecules > 200 nt that are found in the nucleus/cytoplasm and undergo eukaryotic transcription (15). Earlier, lncRNAs were regarded as a byproduct of the transcription of RNAP II and thought to have minimal physiological activity. However, numerous studies have shown that they act as multi-level regulators of protein-coding gene expression, by epigenetic (16, 17), transcriptional (18, 19), and post-transcriptional regulation (20, 21). RNA-seq is effective for identifying differentially expressed (DE) genes as well as quantifying transcriptomes (22, 23). This technique has been frequently used in the functional analysis of lncRNAs/mRNAs in adipose tissue in cattle (24–27), sheep (28), pigs (29), and chickens (30). Jiang et al. (25) isolated 3,716 candidate lncRNAs from fat samples from calves and adult cattle and confirmed their crucial role in cattle of various ages. Liu et al. (26) demonstrated that genes, such as *PLIN1, PLIN2*, and *PPARGC1A* regulated various lipid-related pathways. Cai et al. (31) identified a novel lncRNA, *BADLNCR1*, that suppressed bovine adipogenesis by inhibiting *GLRX5* expression. However, the roles of lncRNA/mRNAs in UFA synthesis in bovine adipocytes are poorly understood. Here, we used RNA-Seq to examine the lncRNAs/mRNA levels in bovine adipocytes by RNA interference and non-interference of *ACSL1*. We screened both lncRNAs and mRNAs in terms of UFA synthesis to understand their role in the regulation of UFA synthesis in bovine adipocytes.

## Materials and Methods

### Ethics Statements

A healthy 1-day-old calf was born and raised at the animal farm of Gansu Agricultural University (LanZhou, China) and humanely slaughtered in a slaughterhouse, followed by the collection of the perirenal adipose tissue along with heart, spleen, kidney, liver, lung, and leg muscle tissues. The study protocol was approved by the Gansu Agricultural University, China, as well as the Ministry of Science and Technology of the People's Republic of China (Approval number 2006–398). Animal experimentation, including sample collection, was performed in agreement with the ethical commission's guidelines of Gansu Agricultural University. Furthermore, the experimental protocol complied with the local animal welfare guidelines.

### Sample Collection and Cell Culture

The perirenal adipose tissue of 1-day-old calf (from the animal farm of Gansu Agricultural University) was collected and shredded in PBS, followed by the addition of type I collagenase (200 U/mL). The solution was shaken at 37°C for 1 h, and enzymatic digestion was stopped by the addition of an equal volume of complete medium. The digested product was serially filtered through 100 μm and 40 μm mesh filters, followed by centrifugation (1,500 rpm, 10 min). The erythrocyte lysate was mixed with the pellet and centrifuged (1,500 rpm, 10 min). After discarding the supernatant, a complete medium was added to the pellet, mixed, and centrifuged twice (1,500 rpm, 10 min). The pellet was resuspended, and the cells were seeded in a 10 cm cell culture dish and cultured in 5% CO_2_ at 37°C. The medium was refreshed after 12 h to remove the non-adherent cells and was refreshed every 48 h. At 90% confluence, the cells were removed with trypsin and frozen in liquid nitrogen.

### *ACSL1* Expression

Bovine pre-adipocytes were grown and passaged to the F3 generation, and cells were isolated on differentiation days 0, 2, 4, 6, and 8. We performed qRT-PCR to assess *ACSL1* expression at different stages of differentiation.

### Synthesis and Screening of *ACSL1* siRNA

The complete sequence of bovine *ACSL1* (GenBank No. 537161) was used to design and synthesize three siRNA sequences (Guangzhou Ruibo Biological Company). Table 1 shows the sequence information.

**Table 1:** Target sequences of siRNAs for bovine *ACSL1*.

**siRNA No**.	**Target sequence**
*ACSL1* siRNA-1	GGATAGAGGAGTACCTGTA
*ACSL1* siRNA-2	CCCTATGAATGGCTTTCAT
*ACSL1 siRNA-3*	ACTCTTCTCTATCGACAAT

Next, the synthesized siRNA-ACSL1 and the corresponding siRNA-Non-specific Control (siRNA-NC) were transfected into bovine adipocytes. After 48 h of culture, we performed qRT-PCR to determine *ACSL1* mRNA expression to screen the siRNA-ACSL1 with the highest interference efficiency.

Next, we sequenced the bovine adipocytes in which *ACSL1* expression was silenced and those that exhibited normal expression.

### RNA Quantification

We used TRIzol reagent to isolate total RNA from siRNA-ACSL1 or siRNA-NC bovine adipocytes, followed by strict quality control of the RNA samples. First, 1% agarose gel electrophoresis (AGE) was used to analyze RNA integrity and the presence of DNA contamination. RNA purity was measured using an IMPLEN NanoPhotometer® spectrophotometer and quantified using an RNA analysis kit and a Qubit®2.0 Fluorometer. Finally, the RNA Nano 6000 Assay Kit was used to assess RNA integrity using the Agilent Bioanalyzer 2100 system.

### Library Preparation for Transcriptome Sequencing

The cDNA library was prepared using the NEBNext Ultra Directional RNA Library Prep Kit for Illumina sequencing using a combination of equal amounts of total RNA of two parallel bovine adipocytes cultures in equal ratios of siRNA-ACSL1/siRNA-NC. The total RNA was from 6 dishes of bovine adipocytes, of which 3 dishes were treated with ACSL1 gene interference, and the other 3 dishes were the control group. The Epicenter Ribo-zero rRNA Removal kit was used for the removal of the rRNA, followed by the construction of six libraries, which were labeled si_ACSL1_1, si_ACSL1_2, si_ACSL1_3, NC_ACSL1_1, NC_ACSL1_2, and NC_ACSL1_3, respectively. Briefly, poly-T oligo-attached magnetic beads were used to obtain mRNA, followed by fragmentation using divalent cations at high temperatures in NEBNext First Strand Synthesis Reaction Buffer (5X). First-strand cDNA was prepared using a random hexamer primer and M-MuLV Reverse Transcriptase (RNaseH-), followed by the application of DNAPI and RNase H. These activities facilitated the conversion of the remaining overhangs into blunt ends. The NEBNext adaptor with hairpin loop structure was ligated to prepare for hybridization after adenylation of the 3′ ends of the DNA fragments. The Beckman Coulter AMPure XP system was used to obtain cDNA fragments of 150 ~ 200 bp in length. These fragments were treated with 3 μL of USER Enzyme at 37°C for 15 min, followed by 5 min at 95°C before PCR. Next, Phusion High-Fidelity DNA polymerase, Universal PCR primers, and Index (X) Primer were used for PCR analysis. Finally, the Agilent Bioanalyzer 2100 system was used to assess the quality of the library generated. The index-coded samples were clustered using a cBot Cluster Generation System for TruSeq PE Cluster Kit v3-cBot-HS (Illumina). An Illumina HiSeq PE150 platform was used to sequence the library at Novogene Bioinformatics Institute (Beijing, China), resulting in the generation of data on the 150 bp paired-end reads.

### Analysis of Sequencing Data

In-house Perl scripts were used to analyze the raw reads. Clean reads were obtained after filtering the raw data for low-quality reads/adaptors/polyNs, and at the same time, we obtained the Q20, Q30, and GC information. The STAR (v2.5.1b) software (32) was used to create a list of the reference genome, and the corresponding paired-end clean reads were aligned to the cattle reference genome (Bos_taurus_Ensembl_94). We used HTSeq v0.6.0 to assess the read counts mapped to each gene. The gene lengths and read counts aligned to the genes were calculated using FPKM. Normalization of the expression of lncRNAs/mRNAs removed the impact of sequencing depth, gene length, and sample difference on gene expression. The R package DESeq2 was used to identify DE-lncRNAs (DELs) and protein-coding genes (33) following a negative binomial distribution. Genes and lncRNAs with an adjusted *P* < 0.05 (*P*-adjust) were labeled as differentially expressed.

### Prediction of lncRNA

Cuffcompare software was used to screen the lncRNA transcripts. The assembled transcripts were filtered to determine probable lncRNAs to reduce the false-positive rate using the following steps: (1) New transcripts were identified by comparison with reference annotations; (2) Transcripts with exon numbers ≥ 2 and lengths > 200 nt were selected for the exon number screening and transcript length screening of the transcripts; (3) Transcripts were assessed for coding potential by integration using coded potential calculator 2 (CPC2) (34), Pfam (35), and Coding-Non-Coding-Index (CNCI) (36) to predict the coding potential and the intersection of the non-coding transcripts, allowing selection of the candidate lncRNA dataset; (4) The candidate novel lncRNAs were finally screened using the HUGO Gene Nomenclature Committee (HGNC) naming guidelines for long non-coding RNA (37) and named based on their positional relationships with coding genes.

### Target Gene Prediction of lncRNAs

The target genes for the lncRNAs in the RNA-seq results were predicted based on cis-and trans-regulation. We investigated the coding genes within 100 kb of the lncRNAs to determine their target genes (38). The trans role implied an expression-based association between the lncRNA and target genes. We determined the correlation between the lncRNAs and coding genes with custom scripts, and the genes with Pearson correlation coefficient > 0.95 were identified as the target genes of the lncRNAs (39).

### GO and KEGG Analyses of the DEMs and lncRNA Target Genes

The GOseq R package was used to perform Gene Ontology (GO) analysis of the target genes of differentially expressed mRNAs (DEMs)/lncRNA target genes, with corrected gene length bias (40). The KEGG database was used to evaluate the functional significance of the biological systems (cells, organisms, and the ecosystem) based on genome sequencing and other high-throughput experimental data. The cluster Profiler R package was used for KEGG pathway analysis of the DEMs/lncRNA target genes. A *P* < 0.05 indicated a significant relationship between the terms and the DE-lncRNA target genes.

### The DEL-DEM Co-expression Network

Next, we obtained the differentially expressed lncRNAs (DELs) and their target genes from the cis and trans prediction results, respectively, to further explore the interactions between the DEMs and DELs. These were incorporated with the DEMs from the sequencing results. We screened the lipogenic-related mRNAs based on literature searches to improve the visibility of the results and finally constructed a DEL-DEM co-expression network using Cytoscape (v3.6.0) (41).

### Experimental Verification of mRNAs and lncRNAs

We randomly selected 12 significant DEMs and 7 DELs and analyzed their expression using qRT-PCR. The Evo-MLV kit was used to synthesize the RNA extracted from bovine adipocytes. First, we used Primer 5.0 to design specific primers for mRNAs and lncRNAs. Tables 2, 3 show the primers of DEMs and DELs used, respectively. The PCR products were analyzed using 1.5% AGE and then sequenced using the Sanger method. Then, we verified the mRNAs/lncRNAs from bovine adipocytes by comparing the amplified sequences of the PCR products with the sequences of the specific mRNAs and lncRNAs obtained from RNA-Seq using MEGA 5.0. Second, the Applied Biosystems QuantStudio®6 Flex was used to perform qRT-PCR using the TB Green® Premix Ex Taq™ II, and the bovine *GAPDH* gene as the internal reference. The 2^−ΔΔCT^ method was used to calculate relative expression with *GAPDH* as the internal control (42). Also, the expression of mRNAs and lncRNAs from both the techniques were expressed as Log_2_ (fold change) for the si-treated group in comparison with the NC-treated group. Paired *t*-tests using GraphPad Prism v6 were used for data analysis. Data were expressed as the mean ± SE deviation of triplicate experiments. A *P* < 0.05 indicated a significant difference.

**Table 2:** mRNAs' primers used in the qRT-PCR.

**mRNAs**	**Forward (5′ → 3′)**	**Reverse (5′ → 3′)**
NELL2	ACAATAGTGGCGACACCTGG	CGTCCAGGCAAGTTTTGGTG
ACSL1	TGCTGCCTGACTGTTGCT	ACCACTTGCCAATGTCCC
SLC26A7	TGGAGTGGGCGACACATTAC	TGACAGAACAGCAAAGGCCA
FABP4	TTCCTTCAAATTGGGCCAGGA	AGTTCGATGCAAACGTCATCC
TMEM87B	AGCCTCGTCTAGGAACCGT	ATCAAGAAGAGACAGAGGGAGG
OLR1	CTTTGTCTGGGATTACTGG	GTGGGCAAGGGTTTCTAT
TGFBI	CCCCGTGGAGAACTGAACAA	ATGTCCACCTCAGCAACAGG
PTGIS	TCCTGGGCCGTGGTCTT	TAGGAGTGGGGATCCAGGAG
VCAN	GATTACGGGTGGCTGTTGGA	GATTACGGGTGGCTGTTGGA
FMOD	ACAGCCATGTACTGCGACAA	TCACTGGTGATCTGGTTGCC
IDH3A	ACCTGTGTGCGGGATTGATT	CTTCGCAGCGTGGTCAAAAA
EMP3	GCCCTCCACATCCTCATT	CTTCAGCCAGCCGTTCTC
GAPDH	AGTTCAACGGCACAGTCAAGG	ACCACATACTCAGCACCAGCA

**Table 3:** lncRNAs' primers used in the qRT-PCR.

**lncRNAs**	**Forward (5′ → 3′)**	**Reverse (5′ → 3′)**
TCONS_00069661	TGCCATTCCTTTCGTTCTTCT	TCCTCTGCTTTCCCACTGTTT
TCONS_00139018	CGTACTCCTTTCCCAATT	TGCCTCCTGAGAAATCTG
TCONS_00057814	TGGGTCTGTGCGTTTGCG	TCTGGTGGAGGTCCGTAGCG
TCONS_00050038	GCCCTGACAACGGCTACCT	TGGGATTCCAGGCCCTTCC
TCONS_00002149	CCTGCCTTGACTGTTTGA	CCTGTTGAGATGCCTCTTT
TCONS_00057808	CACTAGGCACTCGCATTCC	GCAAACGCACAGACCCAC
TCONS_00040771	AAGAGGGCTTTGGAGTGA	TTCTGCCATAAGGGTGGT
GAPDH	AGTTCAACGGCACAGTCAAGG	ACCACATACTCAGCACCAGCA

Next, the lncRNA levels were determined in various bovine tissues to further verify the role of lncRNA in the production of bovine UFAs. Briefly, we used TRIzol to isolate total RNA from the collected heart, spleen, lung, liver, kidney, leg muscle, and perinephric fat, followed by reverse transcription to generate cDNA. Finally, the expression of lncRNAs was determined using qRT-PCR. The 2^−ΔΔCT^ method was used to determine the relative expression using *GAPDH* as the internal control. The paired *t*-test using GraphPad Prism v6 was done for data analysis. Data were expressed as the mean ± SE deviation of triplicate experiments.

## Results

### Temporal Expression of *ACSL1* During the Differentiation of Bovine Adipocytes

The cultured F3 generation bovine pre-adipocytes were collected on differentiation days 0, 2, 4, 6, and 8, and qRT-PCR was performed to determine the relative expression of *ACSL1*. The rise in *ACSL1* levels was followed by a reduction, with the maximum expression on day 4 (*P* < 0.01). Therefore, day-4 bovine adipocytes were used for subsequent experiments (13).

### Screening of Effective siRNA for *ACSL1*

We transfected three pairs of siRNAs into the bovine adipocytes and determined their expression after 48 h to screen the most efficient siRNA fragments for silencing *ACSL1*. There was a >70% reduction in the mRNA expression of *ACSL1*, with si3-ACSL1 showed the highest efficiency of interference (86%; *P* < 0.01). Therefore, si3-ACSL1 was selected for subsequent experiments (13). Next, we transfected differentiation day 4 adipocytes with si3-ACSL1 (si-treated group) (13), followed by sequencing of both the si-treated cells and NC-treated cells.

### Sequencing Data Summary

**Figure 1:**
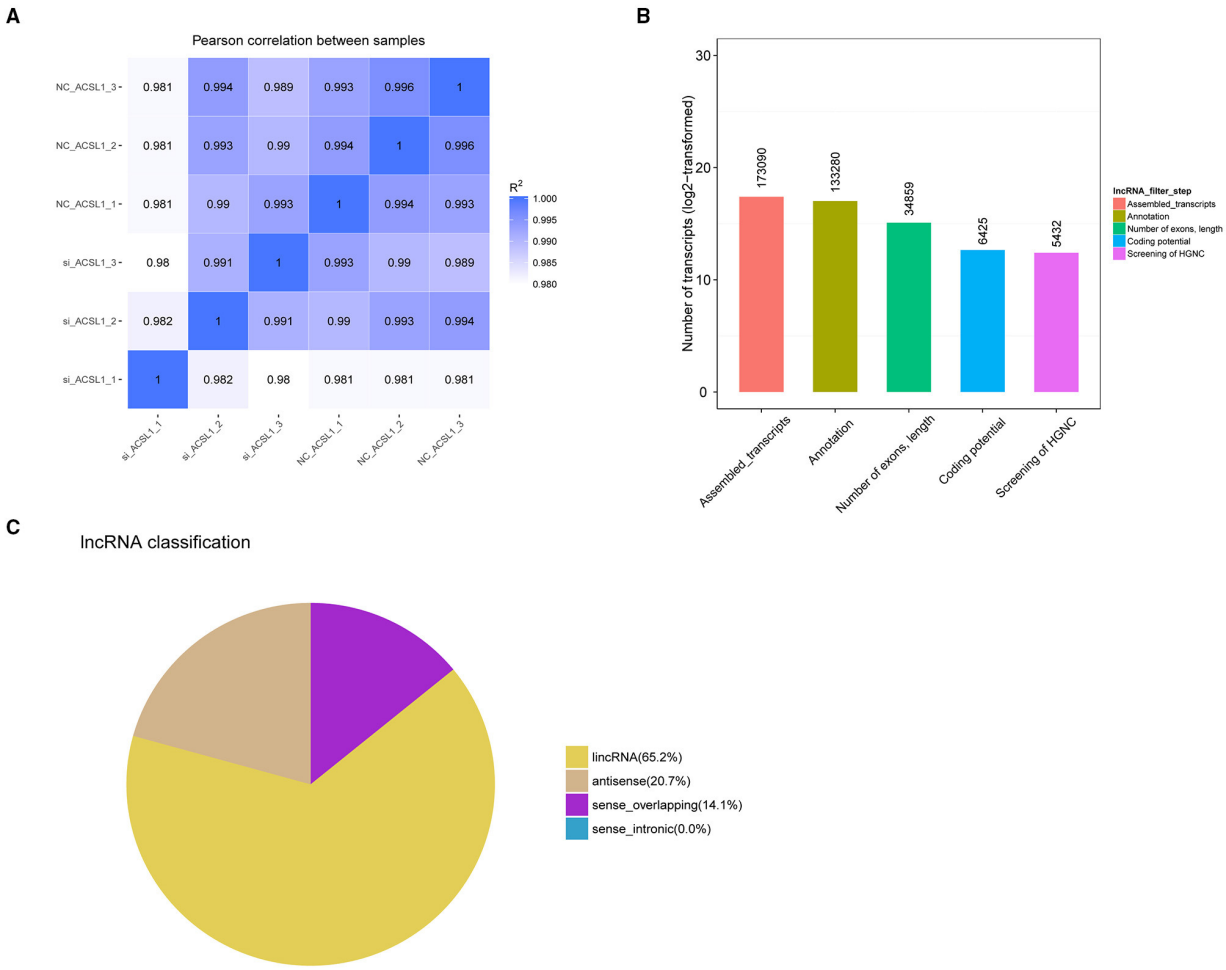
Sequencing data after manipulation of *ACSL1* expression in bovine adipocytes. **(A)** Pearson correlation coefficients between samples. **(B)** LncRNA were screened using Cuffcompare software. **(C)** Classification of lncRNA.

We constructed six cDNA libraries for the NC-treated group (*n* = 3) and si-treated group (*n* = 3) to explore the regulation of UFA synthesis by mRNAs and lncRNAs. After sequencing, the raw reads were acquired using the Illumina HiSeq PE150 Platform and deposited in GenBank (accession numbers: SRR13358757-13358762). Next, we tested the correlation between the expression of different samples (Figure 1A). In total, we obtained 98,462,108–117,507,866 and 93,238,078–110,700, 276 raw reads from the libraries from the NC-treated group and si-treated group, respectively, which resulted in 96,857,744–115,542,046 and 91,106,662–109,206,440 clean reads after removing low-quality/adaptor sequences, resulting in ~15 GB data/sample. The GC contents were 51.54–56.29%. Q20 was >96%, and Q30 was >90% (Table 4). More than 87.76% of clean reads aligned to the reference genome, resulting in 173,090 assembled transcripts (Supplementary File 1). Our study identified 5,432 putative lncRNAs, which included 65.2% lincRNAs, 20.7% antisense lncRNAs, and 14.1% sense_overlapping lncRNAs (Figures 1B,C). At the transcriptional level, 15,547 mRNAs were obtained, of which 454 were novel mRNAs.

**Table 4:** Summary of the sequencing data.

**Sample_name**	**Raw_reads**	**Clean_reads**	**Raw_bases (G)**	**Clean_bases (G)**	**Q20 (%)**	**Q30 (%)**	**GC_content (%)**
NC_ACSL1_1	117,507,866	115,542,046	17.63	17.33	97.49	93.04	55.28
NC_ACSL1_2	98,462,108	96,857,744	14.77	14.53	97.65	93.44	53.35
NC_ACSL1_3	103,447,506	101,411,748	15.52	15.21	97.73	93.64	53.64
si_ACSL1_1	110,700,276	109,206,440	16.61	16.38	97.05	92.25	56.29
si_ACSL1_2	100,037,150	98,632,486	15.01	14.79	97.76	93.68	52.87
si_ACSL1_3	93,238,078	91,106,662	13.99	13.67	96.38	90.86	51.54

### Genomic Expression of lncRNAs and mRNAs

**Figure 2:**
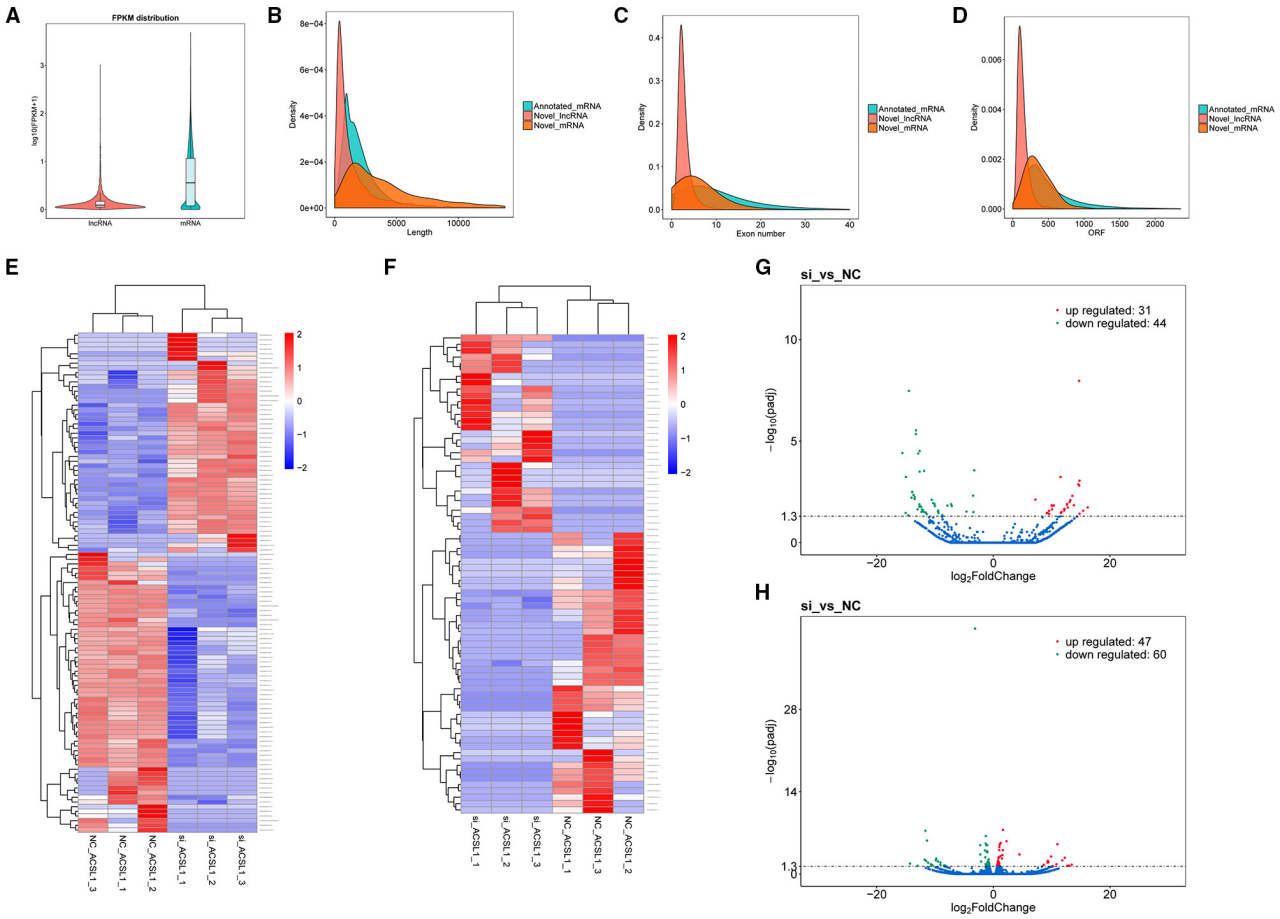
Genomic expression of lncRNAs and mRNAs. **(A)** The expression of lncRNA and mRNA. **(B–D)** Comparison of length, exon count, and ORF length between lncRNAs and mRNAs. **(E,F)** Cluster heatmap of DEMs and DELs. The FPKM array was centered and scaled in the row direction by R package pheatmap (v1.0.8). Red indicates higher expression and blue represents lower expression. The log_10_ (FPKM+1) value was converted (scale number) and clustered. **(G,H)** Volcano plot of DELs and DEMs.

We analyzed the gene structure and expression to determine the dissimilarities between the lncRNAs and mRNAs obtained by RNA-seq. The results showed that mRNAs were expressed at a relatively higher degree with the lncRNAs, based on the FPKM values (Figure 2A). The mRNAs (5,321 bp on average) were substantially longer than the lncRNAs (1,813 bp on average) (Figure 2B). The mRNAs had a higher number of exons (6.04 on average) than those of the lncRNAs (2.91 on average). Also, 61.23% of mRNAs had four or more exons, while 81.41% of lncRNAs had three or fewer exons (Figure 2C). Furthermore, most of the mRNAs (402 bp on average) had longer ORFs than the lncRNAs (147 bp on average) (Figure 2D).

We detected 75 DELs and 107 DEMs in the si-treated group compared with the NC-treated group (*P*-adjusted <0.05). We performed differential mRNA cluster analysis to examine the clustering pattern of DEMs under varying experimental conditions. We found that the same group of DEMs was clustered together (Figure 2E), supporting the accuracy and reliability of the samples. Furthermore, we found that in the si-treated group, 31 lncRNAs were significantly upregulated, and 44 lncRNAs were significantly downregulated (*P*-adjusted <0.05). Figure 2F shows the clustering results. Similarly, we observed significantly upregulated expression of 47 mRNAs and downregulated expression of 60 mRNAs in the si-treated group (*P*-adjusted <0.05). The volcano plots (Figures 2G,H) and the Supplementary Files 2, 3 show the DEL and DEMs, respectively.

### GO and KEGG Analyses of the DEMs

**Figure 3:**
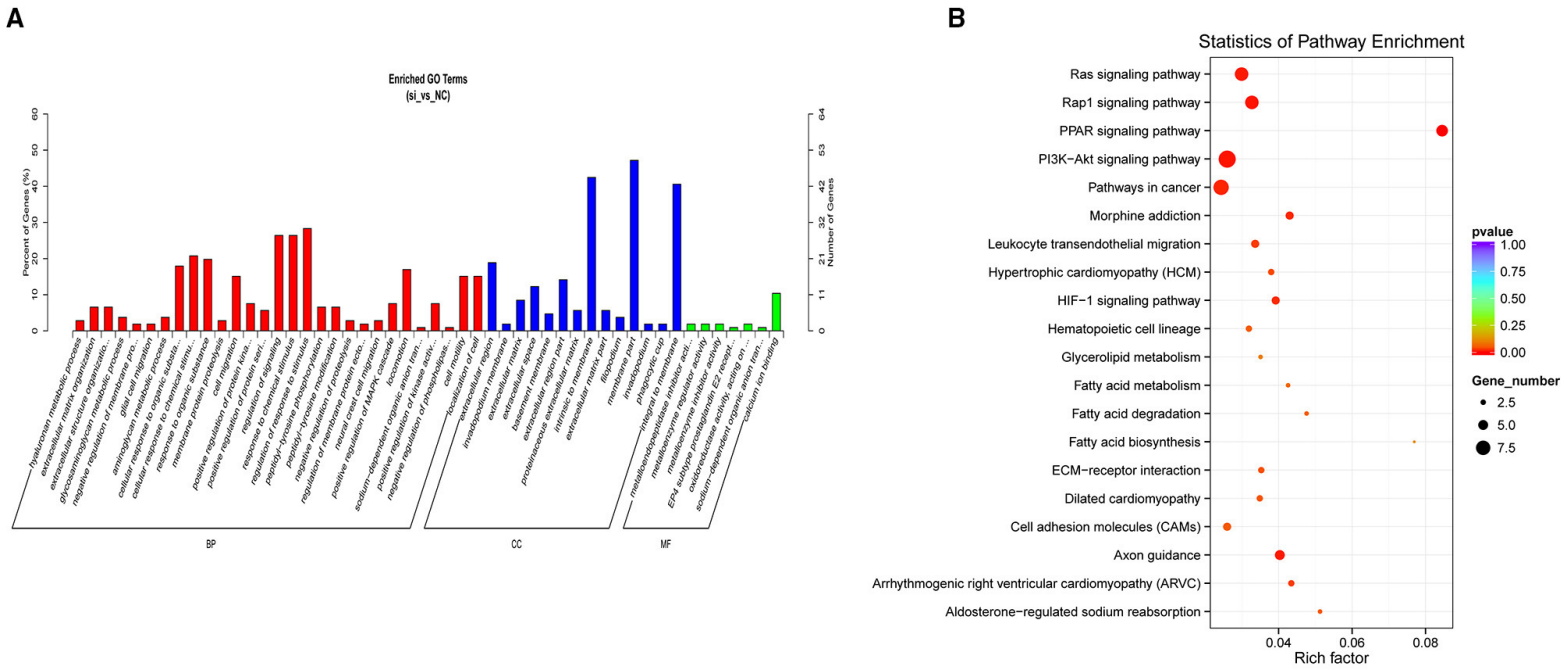
GO and KEGG analysis of DEMs. **(A)** GO analysis of DEMs showing the relevant categories. **(B)** Top 20 of KEGG pathways of DEMs.

The results of the GO analysis revealed that the significant DEMs were classified into 386 functional groups (*P* < 0.05; Supplementary File 4). Of these, 64 terms were significantly enriched in molecular function (GO-MF), and metalloendopeptidase inhibitor activity (GO: 0008191; *P* = 0.0017) was the most significantly enriched GO term, followed by EP4 subtype prostaglandin E2 receptor binding (GO: 0031867; *P* = 0.0019) and oxidoreductase activity (GO: 0016671; *P* = 0.0034). For the cellular component (GO-CC), 33 terms were significantly enriched, with the extracellular region (GO: 0005576; *P* = 0.0001) the most significantly enriched, followed by the invadopodium membrane (GO: 0071438; *P* = 0.0004). For the biological processes (GO-BP), 289 GO terms were related to various processes or regulation, such as glycosaminoglycan metabolic process (GO: 0030203; *P* = 0.0009), aminoglycan metabolic process (GO: 0006022; *P* = 0.0014), and positive regulation of MAPK cascade (GO: 0043410; *P* = 0.0055). Figure 3A shows the top 50 functional GO annotations for the DEMs. The KEGG pathway analysis showed that the DEMs were involved in 116 signaling pathways (Supplementary File 5). Figure 3B shows the top 20 signaling pathways, and the PPAR signaling pathway showed the highest level of significance (*P* < 0.05), followed by the Rap1 signaling pathway and PI3K-Akt signaling pathway. Our results indicated that these pathways were probably involved in UFA synthesis.

### Cis-/Trans-Regulation of the lncRNAs Target Genes

**Figure 4:**
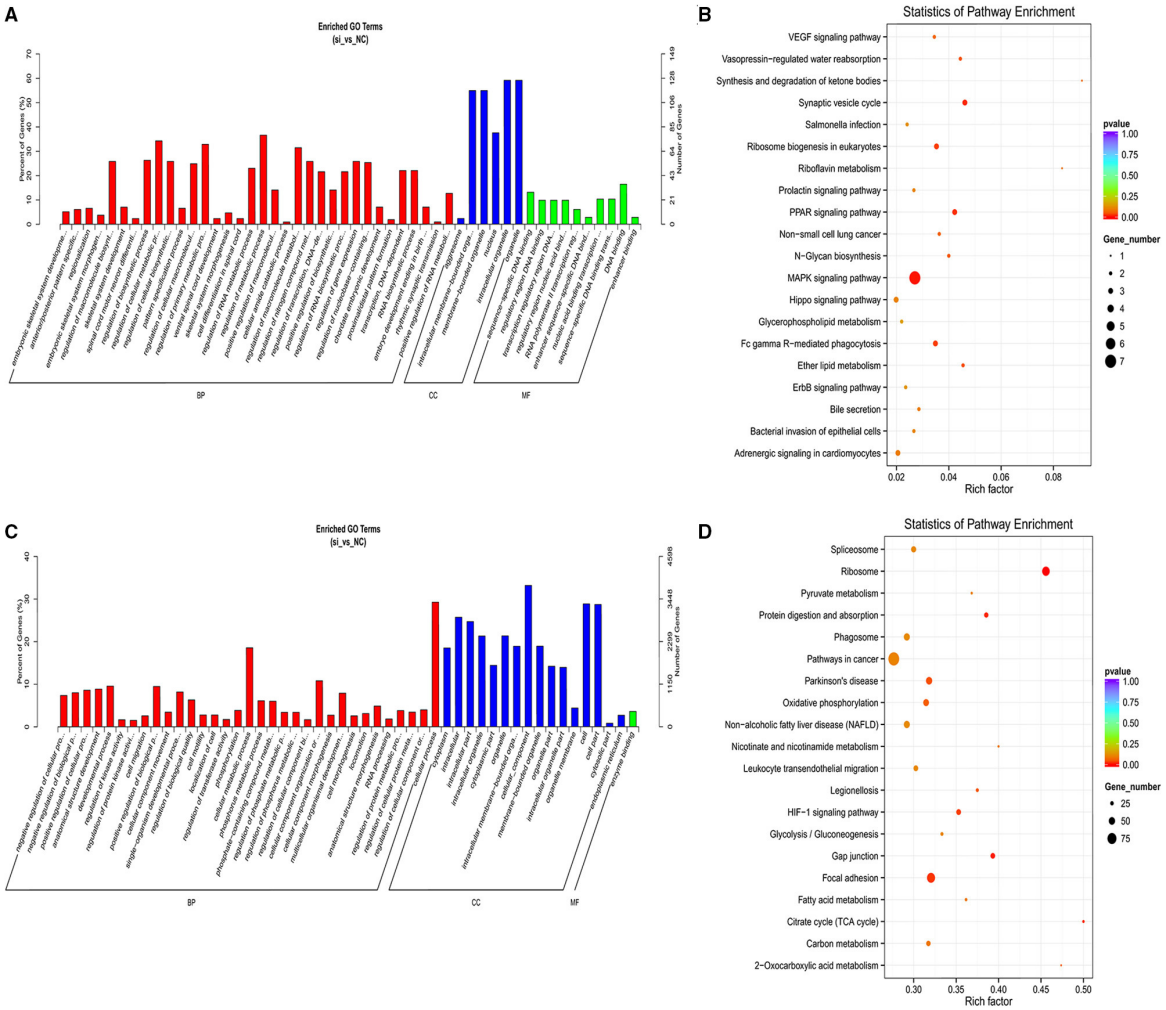
GO and KEGG analyses of cis and trans target genes of DELs. **(A)** The GO analysis of top 50 cis target genes with the corresponding categories. **(B)** Top 20 of KEGG pathways analysis of cis target genes. **(C)** Top 50 GO analysis of trans target genes along with the categories of BP, CC, and MF. **(D)** The KEGG pathways of the top 20 trans target genes.

We identified 20,724 protein-coding genes as the nearest neighbors of 5,423 out of 5,432 lncRNAs using 100 kb as the cutoff (Supplementary File 6). Subsequent GO analysis revealed 435 significantly enriched GO terms (*P* < 0.05; Supplementary File 7). Figure 4A shows the top 50 GO functional annotations of the DEL target genes. The top five GO terms were embryonic skeletal system development, anterior/posterior pattern specification, sequence-specific DNA binding, regionalization, and embryonic skeletal system morphogenesis. The results of KEGG analysis indicated the involvement of seven pathways (*P* < 0.05; Supplementary File 8), and Figure 4B shows the top 20 KEGG pathways, such as MAPK signaling pathway, PPAR signaling pathway, Ribosome biogenesis in eukaryotes, and ether lipid metabolism.

Next, we identified 5,432 lncRNAs and 698,719 genes to investigate the trans target genes of lncRNAs using the Pearson correlation ≥ 0.95 as the cutoff (Supplementary File 9). Figure 4C shows the top 50 GO functional annotations of the DEL target genes. GO analysis identified 1,154 significantly enriched GO terms (Supplementary File 10), and the top five GO terms were enzyme binding, kinase regulator activity, rRNA binding, G-protein coupled nucleotide receptor activity, and G-protein coupled purinergic nucleotide receptor activity. We also found that 274 KEGG pathways were involved in the trans-target genes for lncRNAs (Supplementary File 11), such as the Ribosome, Citrate cycle (TCA cycle), protein digestion and absorption, FA metabolism, and FA degradation (Figure 4D).

### Network Construction Based on DEMs and DELs

**Figure 5:**
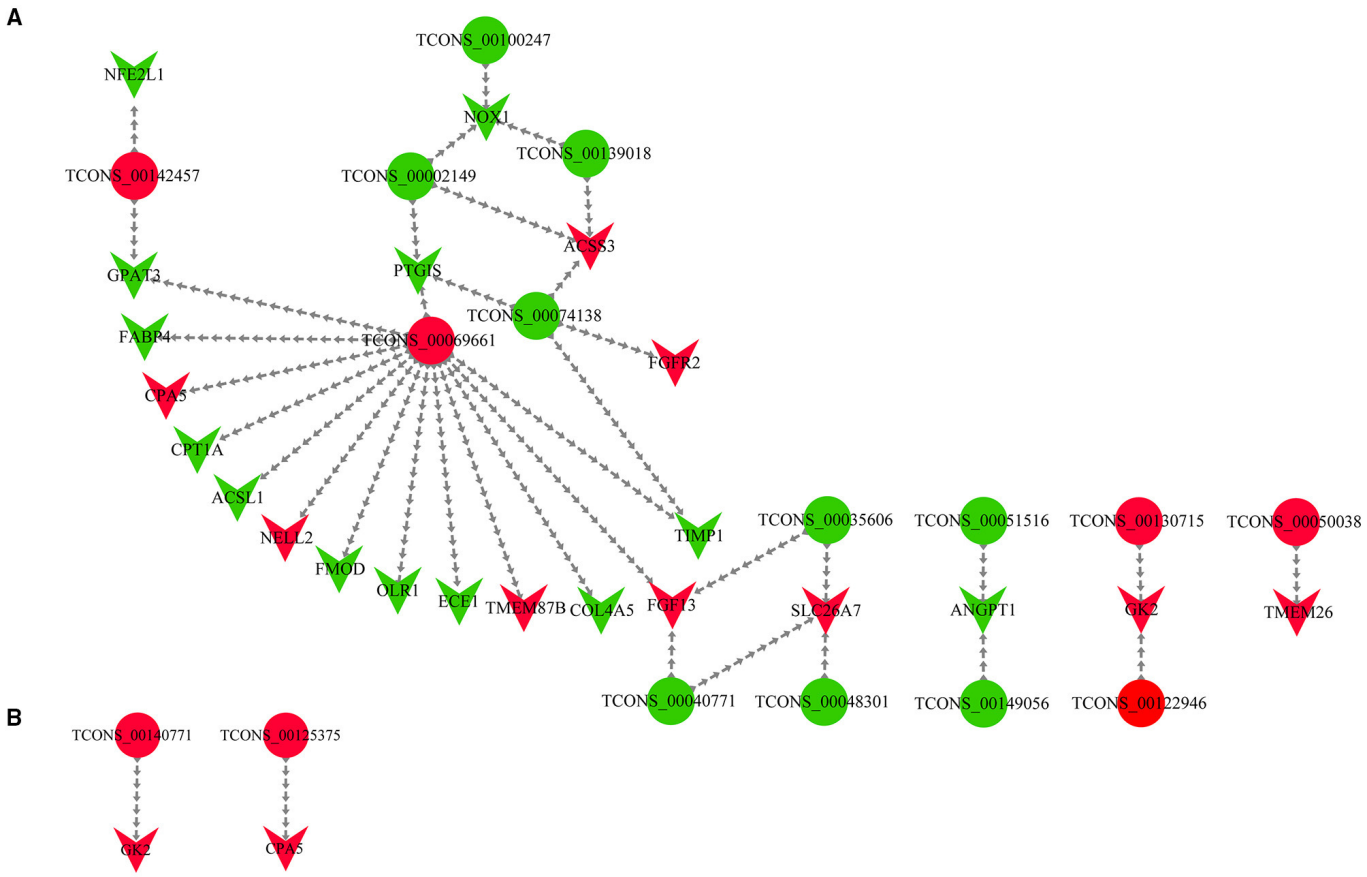
The DEL-DEM interaction network. **(A)** DEL and trans-target interaction network. The trans-target was a DEM as well. **(B)** DEL and cis-target interaction network. The cis-target was a DEM as well. The circle type and inverted triangles type represent a lncRNA and mRNA, respectively. Red represents upregulation, and the green represents downregulation.

We obtained the DELs and their targets from the cis and trans prediction results, respectively, and incorporated them with the DEMs from the sequencing results (Supplementary File 12). Then, we screened lipogenic-related mRNAs based on literature searches and finally constructed a DEL-DEM co-expression network (Supplementary File 13). The DEL-DEM co-expression network involved 14 DELs and 22 trans-targets (Figure 5A), as well as 2 DELs and 2 cis-targets (Figure 5B). The co-expression network results showed that some lncRNAs interacted with multiple DEMs; for example, 14 DEMs co-expressed with *TCONS_00069661*, and 2 DEMs co-expressed with *TCONS_00142457, TCONS_00040771*, and *TCONS_00074138*, respectively, indicating that these lncRNAs and mRNAs belonged to the core non-coding/coding RNAs and had important regulatory effects on the synthesis of UFAs.

### qRT-PCR of RNA-Seq Data

**Figure 6:**
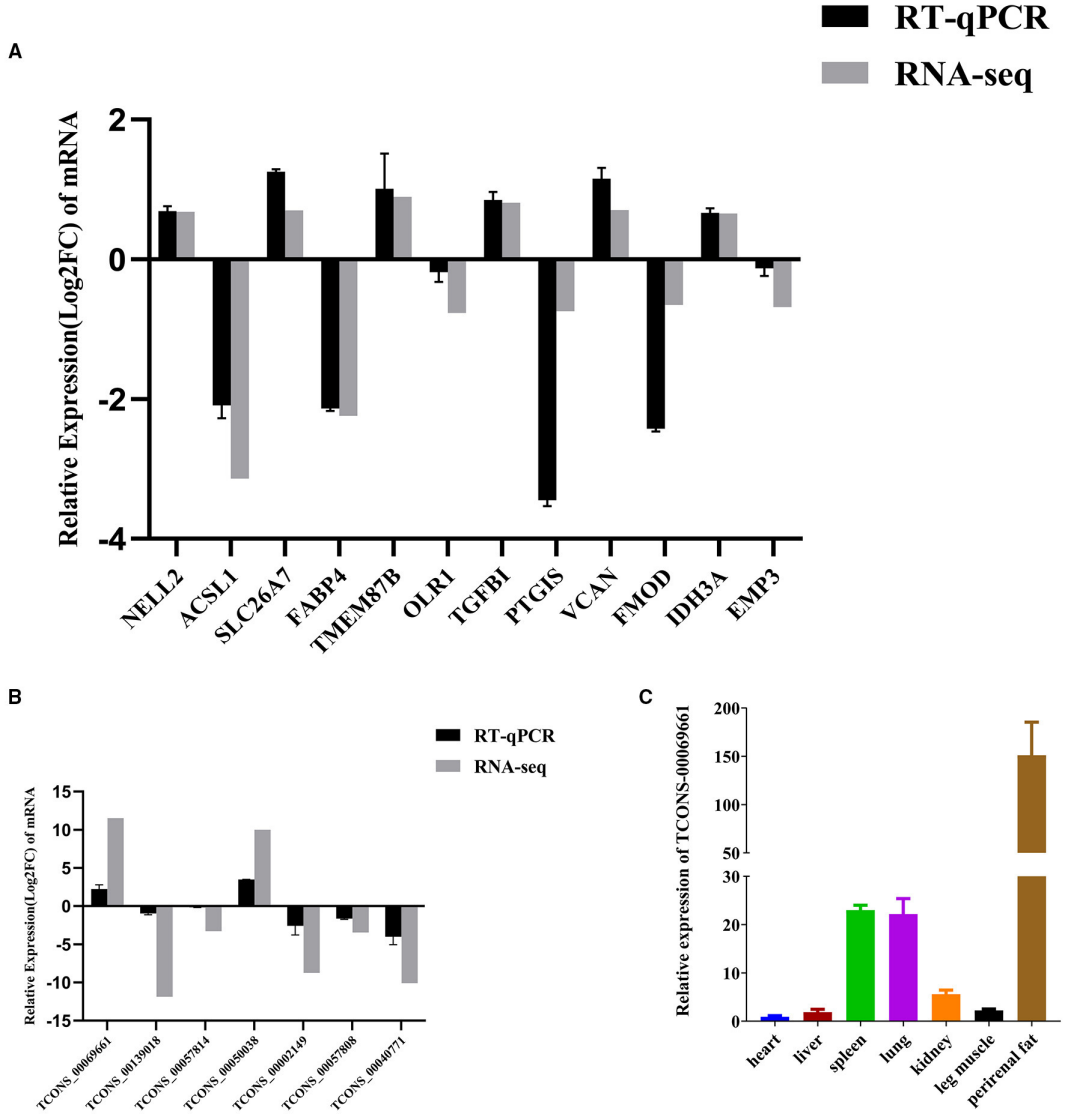
qRT-PCR of DEMs and DELs. **(A)** Changes in mRNA expression between the NC and si-treated groups. **(B)** qRT-PCR verification of changes in lncRNA expression between the NC and si-treated groups. **(C)** qRT-PCR analysis of DEL *TCONS-00069661* in heart, liver, spleen, lung, kidney, leg muscles, and perirenal fat. Three biological and technical replicates were used for each group. The qRT-PCR data were determined using the 2^−ΔΔCt^ method with *GAPDH* as the internal reference. The verification data of mRNA and lncRNA sequencing results were further normalized to log_2_ (foldchange). Data represent means ± standard error.

We randomly chose 12 and 7 genes from DEMs and DELs, respectively, to analyze the validity of the RNA-seq data by qRT-PCR. The results were comparable to the results of mRNA and lncRNA expression derived from the RNA-seq data (Figures 6A,B). Furthermore, the sequence of the qRT-PCR-amplified fragments of the specific primers were consistent with the sequences obtained by RNA-seq (Supplementary File 14).

We used qRT-PCR to study the expression of lncRNA *TCONS_00069661*, located at the center of the co-expression network and expressed in the kidney, spleen, heart, liver, lung, leg muscle, and perirenal fat of cattle. We found that *TCONS_00069661* was highly expressed in the adipose tissue of cattle (Figure 6C). Moreover, our previous studies showed that *ACSL1* gene was also highly expressed in bovine adipose tissue (12). Thus, these results substantiated the reliability of the co-expression network, and suggested the regulatory roles played by DELs and DEMs in UFA synthesis.

## Discussion

In recent years, transcriptome sequencing has been frequently used for gene expression analysis and to reveal biological characteristics. Here, we investigated the expression of *ACSL1*, a key gene involved in the production of UFAs in adipocytes, to explore the molecular mechanism of UFAs synthesis. After different manipulations of *ACSL1*, we performed strand-specific RNA sequencing to identify mRNAs and lncRNAs in bovine adipocytes systematically.

In this study, 15,547 mRNAs and 5,432 lncRNA transcripts were identified in the NC-treated group and si-treated group, of which 107 mRNAs and 75 lncRNA transcripts were differentially expressed, respectively. The qRT-PCR results confirmed the strong correlation between lncRNA and mRNA expression and the transcriptome data. Compared with other transcriptomic studies in beef cattle, fewer mRNAs and lncRNAs were identified in this study. We speculated that it was probably because our study focused specifically on bovine adipocytes. Most of the current research on beef cattle has used specific tissues for sequencing; however, the results were not sufficiently targeted (24, 43). Fewer exons were tested within the 5,432 lncRNAs identified here, which had a lower expression and shorter lengths compared to those of mRNAs. Our results are consistent with the results of other recent studies in other mammals (24, 28, 44). These results on lncRNA expression suggest conservative regulation in mammals.

Next, GO and KEGG analyses were used for determining the biological functions of the mRNAs identified by our sequencing data. The GO analysis classified the significantly enriched DEMs into 386 functional groups (including 289 BPs (*P* < 0.05), 33 CCs, and 64 MFs). Some GO terms were closely related to the production of UFAs and fats, such as positive regulation of MAPK cascade (GO: 0043410; *P* = 0.00545), adiponectin-mediated signaling pathway (GO: 0033211; *P* = 0.025288), long-chain FA transport (GO: 0015909; *P* = 0.030592), and positive regulation of PPAR signaling pathway (GO: 0035360; *P* = 0.031822).

MAPK signaling pathways have been shown to control various biological processes via various cellular mechanisms and might involve activation/inhibition of the molecules involved (45). Wu et al. identified C1qTNF-related factor as one of the adipokines Protein 6 (*CTRP6*), which promoted the differentiation of subcutaneous fat in pigs through the MAPK signaling pathway (46). Adiponectin (*APN*) is a hormone protein that is specifically secreted from human and animal adipose tissue and plays an important role in adipogenesis (47, 48). Only after specific binding of adiponectin to its receptor, can it activate the intracellular signaling cascade to exert its biological effects (49). Additionally, *ADIPOQ* is also known to play a critical role in adipocyte development, which affects the composition of UFAs and lipid production (50). The GO analysis also identified various candidate genes, several of which were demonstrated to be related to UFA synthesis, such as *ACSL1, FABP4*, and *PTGIS*. Additionally, the positive regulation of the MAPK cascade identified eight background genes, including *BMPER, FGFR2*, and *CSPG4*. Although they are not marker genes of UFA synthesis, the regulatory effect of MAPK on adipogenesis indicated that they were probably potential candidates for UFA synthesis.

The results of the KEGG pathway analysis showed that the DEMs were substantially enriched in 13 pathways (*P* < 0.05). Some DEMs were considerably enriched in the PPAR signaling pathway, PI3K-Akt signaling pathway, Rap1 signaling pathway, Ras signaling pathway, and ECM-receptor interaction. The PPAR signaling pathway is a crucial pathway closely related to FA and sterol metabolism as well as adipogenic differentiation (51). The PPAR family involves three subtypes *PPAR-*α/-β*/-*δ/-γ, with variable roles (52). PPARα activates FA oxidation; *PLIN1, PLIN3, LIPE*, and *FABP4* are the known targets of *PPAR* (53). FA-binding protein 4 (*FABP4*) has the ability to predict marbling (54). *PPAR*β*/*δ participates in adipogenic differentiation and adipogenesis (52). In this study, the PPAR signaling pathway was significantly enriched with 6 DEMs: *ACSL1, CPT1A, FABP4, GK2, OLR1*, and *PTGIS*. These genes were all known to be essential genes for lipid production (1, 55).

The PI3K-Akt signaling pathway is a classic insulin-related signaling pathway (56). PI3Ks are involved in the phosphorylation of proteins and the PI3K-Akt pathway might also be involved in adipogenesis (57–59). The insulin-mediated PI3K/AKT signaling pathway plays a vital role within adipocytes of obese patients and leads to an excess of lipids that has to be appropriately stored in the fat tissue (57). In the present study, *ANGPT1, COL4A5, EFNA5, FGF13, FGFR2, ITGA3, ITGA4, KITLG*, and *XLOC_000377* were all considerably enriched in the PI3K-Akt signaling pathway, suggesting that differential expression of these genes might induce the FA-related metabolism pathway in beef cattle.

Rap1, a small G protein, is known to be involved in regulating cell survival/proliferation via the regulation of the PI3-AKT pathway (60). Studies have shown that Ras proteins act as molecular switches to play various regulatory functions in cell growth and survival (61). The Ras signaling pathway has been shown to activate the MAPK cascade; also, the PI3K-Akt pathway is known to regulate cell growth and apoptosis (60). We obtained a total of 14 DEMs from the Rap1 and Ras signaling pathways, which were observed to play vital roles in UFA synthesis. The ECM is essential for tissue architecture and has an important role in adipogenesis (62). ECM is substantially enriched in bovine adipose tissue and may regulate specific genes to participate in adipogenesis (63). In this study, the ECM-receptor interaction was substantially enriched in 3 DEMs, including *ITGA3, ITGA4*, and *COL4A5*. These three genes were more closely related to diseases (64–66), but studies have found that *ITGA3, ITGA4*, and *COL4A1* may be involved in the composition of adipocyte extracellular matrix and the differentiation of adipocytes (67).

For a more in-depth understanding of the DELs potential function, we performed GO and KEGG analyses of its cis and trans target genes. In this study, we identified 75 DELs and predicted their target genes, which were primarily enriched in the GO terms associated with the regulation of FA biosynthesis and metabolism (*P* < 0.05), such as glycerol-3-phosphate biosynthetic process (GO: 0046167), negative regulation of plasma membrane long-chain FA transport (GO: 0010748), FA catabolic process (GO: 0009062), lipid oxidation (GO: 0034440), and FA oxidation (GO: 0019395). These findings suggested that the DELs' target genes are involved in functions from the synthesis to metabolism of FAs, indicating the influence the DELs may have on the synthesis and metabolism of UFAs and lipid. Similarly, some lncRNAs are known to play important roles in lipid synthesis in cattle (25, 31, 68). The KEGG analysis results of the target genes of DELs also showed significant involvement (*P* < 0.05) of pathways related to UFA synthesis, such as the PPAR signaling pathway, MAPK signaling pathway, Ether lipid metabolism, and Citrate cycle (TCA cycle). The TCA cycle involves various critical metabolic steps, and ether lipid metabolism is another vital FA metabolism-related pathway (69, 70).

To improve the screening out of lncRNAs and mRNAs that may be related to UFA synthesis more intuitively and in-depth, we constructed a DEL-DEM co-expression network. The DEL-DEM co-expression network involved 14 DELs and 22 trans-targets, as well as two DELs and two cis-targets to demonstrate their potential in UFAs production. In the results of the co-expression network, lncRNA TCONS_00069661 was of particular interest, as it was predicted to simultaneously target 14 DEMs including *ACSL1*, such as *FABP4, OLR1*, and *COL4A5*. We found that *FABP4* was a downregulated DEM, which not only regulated the differentiation of bovine adipocytes in the PPAR signaling pathway and supported the transport of extracellular FAs but was also involved in lipolysis and FA transport in different tissues (71, 72). A recent study on the *FABP4* gene of Yanbian cattle showed that a SNP in *FABP4* substantially affected the fat content, protein content, and marbling level (73). Li et al. (74) found that with increasing age, the concentration of SFA in Yanbian cattle decreased, and the concentration of MUFA increased proportionally with an increase in the percentage of intramuscular lipids leading, in turn, to an increase in the expression of the *FABP4* gene. A SNP (g.8232C > A) in the oxidized low-density lipoprotein receptor 1 (*OLR1*) has been shown to be related to rump fat thickness and weaning weight in Nelore cattle (75–78), indicating its importance in regulating fat deposition. In our study, the upregulated lncRNA *TCONS_00069661* was predicted to target the downregulated gene *OLR1*. We speculate that *TCONS_00069661* targets the 3'-UTR of *OLR1*, thereby preventing its translation. Current research on *COL4A5* has been related to disease, and some of the genes in this family have been studied in cattle. Previous studies have confirmed the role of *COL5A3, COL6A2*, and *COL3A1* in inducing *CD44* expression, followed by upregulation of the cellular expression of docosanoic acid, palmitic acid, and trans-oleic acid and downregulation of the cellular expression of tridecanoic acid, stearic acid, and cis-5, 8, 11, 14-EPA (79). Liu et al. (26) found that *COL6A1, COL6A2, COL4A2, COL1A1, COL4A6, COL4A5, COL6A3, COL1A2*, and other genes were strongly related to fat metabolism in the transcriptomic study of the muscles of Shandong black cattle and Luxi cattle.

Additionally, our co-expression network results showed that not only did one lncRNA target multiple mRNAs but multiple lncRNAs targeted one mRNA. For example, *TCONS_00040771, TCONS_00035606*, and *TCONS_00048301* simultaneously targeted *SLC26A7*, and *TCONS_00130715* and *TCONS_00122946* simultaneously targeted *GK2*. However, our results of the co-expression network diagram showed dissimilar upward and downward trends of lncRNAs and mRNAs. Thus, we speculate that there is a direct relation between some lncRNAs and mRNAs (21, 31), with some combining with miRNAs to perform the function of ceRNA, leading to the degradation of mRNA targets or translational repression, and the mode of action involved lncRNA-miRNA-mRNA network (80, 81). Further studies should involve the verification of the lncRNA-mRNA targeting relationship pairs that might exist in subsequent studies to explore their mode of action and specific roles in the synthesis of UFAs.

## Conclusion

This study used RNA-seq to determine lncRNA and mRNA levels by interference and comparing *ACSL1* expression with controls in bovine adipocytes. GO and KEGG analyses showed that the target genes of the DELs and DE-genes were enriched in the relevant FA and lipogenesis-related pathways. Based on the above results, we constructed a DEL-DEM interaction network. The results of this study expand our knowledge of the molecular mechanisms used by lncRNAs as well as the genes involved in the regulation of UFA synthesis in bovine adipocytes.

## Data Availability Statement

The datasets presented in this study can be found in online repositories. The names of the repository/repositories and accession number(s) can be found in the article/Supplementary Material.

## Ethics Statement

The animal study was reviewed and approved by Ethical Commission of Gansu Agricultural University as well as the Ministry of Science and Technology of the People's Republic of China.

## Author Contributions

YB, HT, and ZZ: conceptualization. YB, HT, BS, and ZZ: methodology. YB, XLi, JL, YM, and ZC: validation. YB, BS, XLiu, and SL: formal analysis. ZZ, HT, and YB: investigation. YL, JH, and JW: resources. YB: writing—original draft preparation. YB, ZZ, and SR: writing—review and editing. ZZ and XH: supervision. ZZ: project administration. ZZ and JH: funding acquisition. All authors have read and agreed to the published version of the manuscript.

## Funding

This research was supported by the National Natural Science Foundation (31860631), Young Doctor Fund Project of Gansu Provincial Department of Education (2021QB-027), and Scientific research start-up funds for openly-recruited doctors (GSAU-RCZX201711).

## Conflict of Interest

The authors declare that the research was conducted in the absence of any commercial or financial relationships that could be construed as a potential conflict of interest.

## Publisher's Note

All claims expressed in this article are solely those of the authors and do not necessarily represent those of their affiliated organizations, or those of the publisher, the editors and the reviewers. Any product that may be evaluated in this article, or claim that may be made by its manufacturer, is not guaranteed or endorsed by the publisher.

## Supplementary Material

Supplementary File 1 The sequencing results were compared to the reference genome.

Supplementary Files 2, 3 Details of DELs and DEMs.

Supplementary File 4 Details of GO analysis of DEMs.

Supplementary File 5 Details of KEGG pathway analysis of DEMs.

Supplementary File 6 Cis-regulation of the lncRNAs target genes.

Supplementary File 7 GO analysis of DELs cis-regulated target genes.

Supplementary File 8 KEGG pathway analysis of DELs cis-regulated target genes.

Supplementary File 9 Trans-regulation of the lncRNAs target genes.

Supplementary File 10 GO analysis of DELs trans-regulated target genes.

Supplementary File 11 KEGG pathway analysis of DELs trans-regulated target genes.

Supplementary File 12 The cis-regulated target genes and the trans-regulated target genes of DELs intersect with DEMs, respectively.

Supplementary File 13 Literature screening information on lipogenesis-related mRNAs.

Supplementary File 14 The result of the alignment between the sequence of the amplified fragments of qRT-PCR and the sequence in RNA-Seq.
