# Supplementary figures and images for: Interference With ACSL1 Gene in Bovine Adipocytes: Transcriptome Profiling of mRNA and lncRNA Related to Unsaturated Fatty Acid Synthesis
Source: Front Vet Sci. 2021 Dec 16;8:788316. doi: 10.3389/fvets.2021.788316 (PMC8716587; doi:10.3389/fvets.2021.788316)

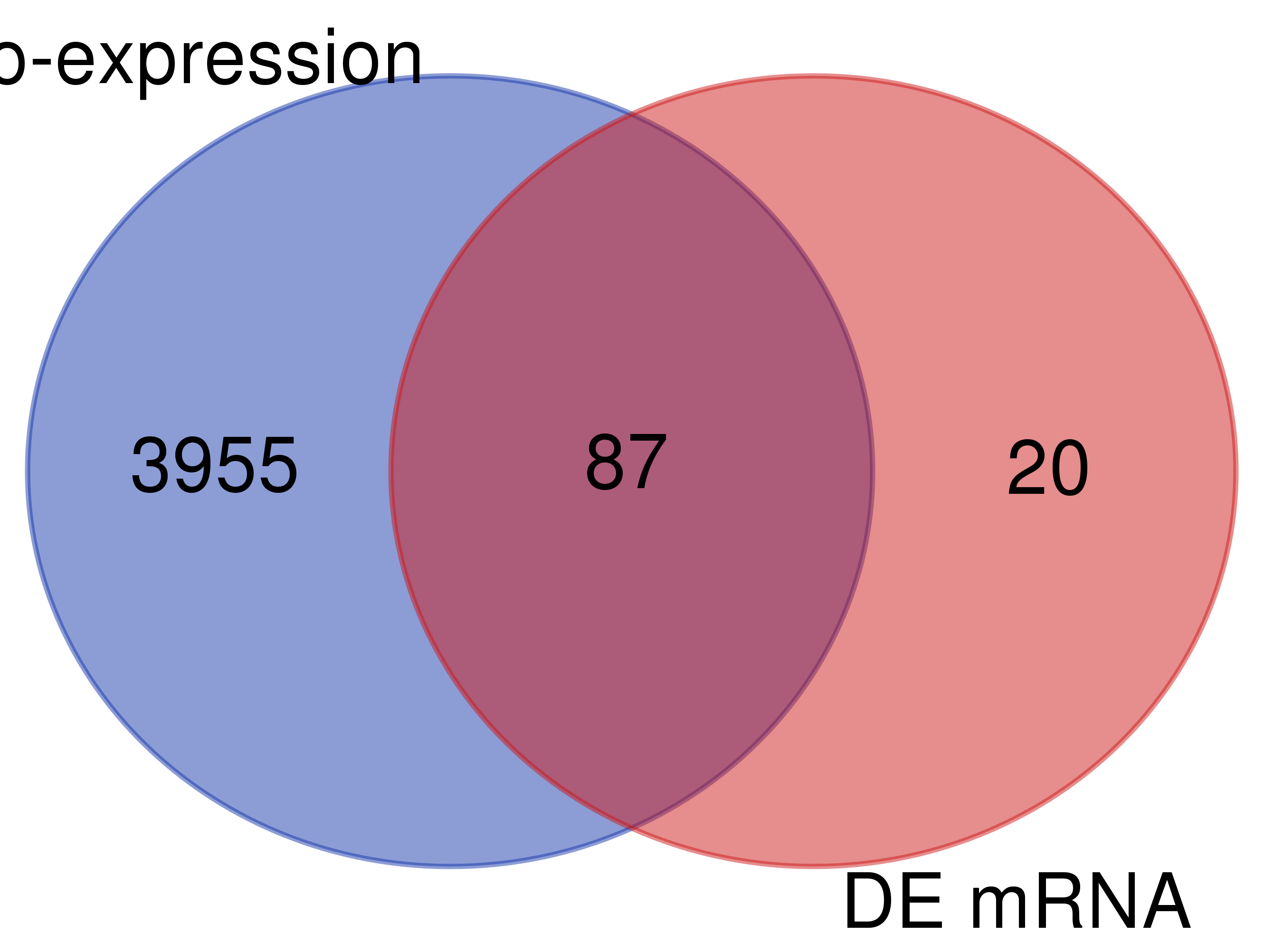

Supplement: Supplementary File 12 — The cis-regulated target genes and the trans-regulated target genes of DELs intersect with DEMs, respectively. [file Data_Sheet_2.ZIP › Supplementary file 12/co-expression---DE mRNA/venn(co-expression---DE mRNA).png]

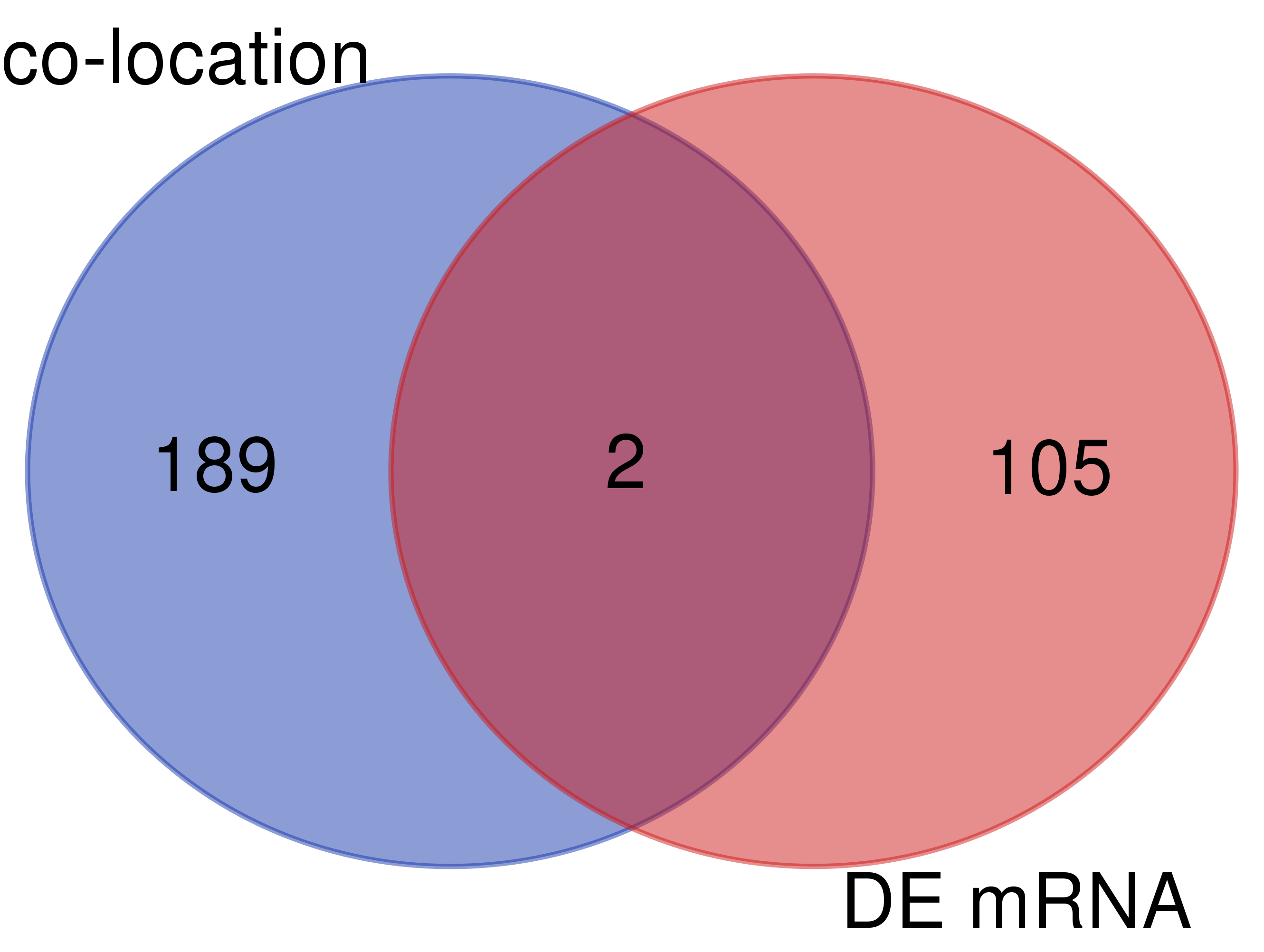

Supplement: Supplementary File 12 — The cis-regulated target genes and the trans-regulated target genes of DELs intersect with DEMs, respectively. [file Data_Sheet_2.ZIP › Supplementary file 12/co-location---DE mRNA/venn(co-location---DE mRNA).png]
