# Supplementary material for: Interference With ACSL1 Gene in Bovine Adipocytes: Transcriptome Profiling of mRNA and lncRNA Related to Unsaturated Fatty Acid Synthesis
Source: Front Vet Sci. 2021 Dec 16;8:788316. doi: 10.3389/fvets.2021.788316 (PMC8716587; doi:10.3389/fvets.2021.788316)
Supplement: Supplementary file 14 [file Table_11.DOCX]

*ACSL1*：

Zhao, Z., Abbas, R. S., Tian, H., Shi, B., Luo, Y., and Wang, J., et al. (2020). Effects of overexpression of acsl1 gene on the synthesis of unsaturated fatty acids in adipocytes of bovine. *Arch. Biochem. Biophys.* 695, 108648. doi: 10.1016/j.abb.2020.108648

*FABP4*:

Gaudel, C., Schwartz, C., Giordano, C., Abumrad, N. A., Grimaldi, P. A. (2008). Pharmacological activation of pparbeta promotes rapid and calcineurin-dependent fiber remodeling and angiogenesis in mouse skeletal muscle. *Am J Physiol Endocrinol Metab*. 295, E297-E304. doi: 10.1152/ajpendo.00581.2007

*CPT1A, GK2*, *PTGIS*:

Chmurzynska, A. (2006). The multigene family of fatty acid-binding proteins (fabps): function, structure and polymorphism. *J. Appl. Genet.* 47, 39-48. doi: 10.1007/BF03194597

Coleman, R. A., Lewin, T. M., Muoio, D. M. (2000). Physiological and nutritional regulation of enzymes of triacylglycerol synthesis. *Annu. Rev. Nutr.* 20, 77-103. doi: 10.1146/annurev.nutr.20.1.77

*OLR1*:

Chen, K. C., Hsieh, I. C., Hsi, E., Wang, Y. S., Dai, C. Y., and Chou, W. W., et al. (2011). Negative feedback regulation between microrna let-7g and the oxldl receptor lox-1. *J. Cell Sci.* 124, 4115-4124. doi: 10.1242/jcs.092767

Fonseca, P. D., de Souza, F. R., de Camargo, G. M., Gil, F. M., Cardoso, D. F., and Zetouni, L., et al. (2015). Association of adipoq, olr1 and ppargc1a gene polymorphisms with growth and carcass traits in nelore cattle. *Meta Gene*. 4, 1-7. doi: 10.1016/j.mgene.2015.02.001

Kaneda, M., Lin, B. Z., Sasazaki, S., Oyama, K., Mannen, H. (2011). Allele frequencies of gene polymorphisms related to economic traits in bos taurus and bos indicus cattle breeds. *Anim. Sci. J.* 82, 717-721. doi: 10.1111/j.1740-0929.2011.00910.x

*COL4A5:*

Hu, Z. L., Park, C. A., Reecy, J. M. (2016). Developmental progress and current status of the animal qtldb. *Nucleic Acids Res.* 44, D827-D833. doi: 10.1093/nar/gkv1233

Liu, R., Liu, X., Bai, X., Xiao, C., Dong, Y. (2020). Different expression of lipid metabolism-related genes in shandong black cattle and luxi cattle based on transcriptome analysis. *Sci Rep*. 10, 21915. doi: 10.1038/s41598-020-79086-4
